# Roles and regulation of autophagy and apoptosis in the remodelling of the lepidopteran midgut epithelium during metamorphosis

**DOI:** 10.1038/srep32939

**Published:** 2016-09-09

**Authors:** Davide Romanelli, Morena Casartelli, Silvia Cappellozza, Magda de Eguileor, Gianluca Tettamanti

**Affiliations:** 1 Department of Biotechnology and Life Sciences, University of Insubria, 21100 Varese, Italy; 2 Department of Biosciences, University of Milano, 20133 Milano, Italy; 3 CREA – Honey Bee and Silkworm Research Unit, Padua seat, 35143 Padova, Italy

## Abstract

We previously showed that autophagy and apoptosis occur in the removal of the lepidopteran larval midgut during metamorphosis. However, their roles in this context and the molecular pathways underlying their activation and regulation were only hypothesized. The results of the present study better clarify the timing of the activation of these two processes: autophagic and apoptotic genes are transcribed at the beginning of metamorphosis, but apoptosis intervenes after autophagy. To investigate the mechanisms that promote the activation of autophagy and apoptosis, we designed a set of experiments based on injections of 20-hydroxyecdysone (20E). Our data demonstrate that autophagy is induced at the end of the last larval stage by the 20E commitment peak, while the onset of apoptosis occurs concomitantly with the 20E metamorphic peak. By impairing autophagic flux, the midgut epithelium degenerated faster, and higher caspase activity was observed compared to controls, whereas inhibiting caspase activation caused a severe delay in epithelial degeneration. Our data demonstrate that autophagy plays a pro-survival function in the silkworm midgut during metamorphosis, while apoptosis is the major process that drives the demise of the epithelium. The evidence collected in this study seems to exclude the occurrence of autophagic cell death in this setting.

In the 1960s, Lockshin and Williams introduced the term “autophagic cell death” and its classification as type 2 cell death while studying the metamorphic degeneration of intersegmental muscles in silkmoths^1^,^2^. Since then, autophagic features have been described in many other larval organs that, in Lepidoptera, are slightly or extensively remodelled during metamorphosis, such as silk glands, fat body, and the midgut^3^,^4^,^5^,^6^,^7^,^8^,^9^,^10^. Following initial studies, which focused on identifying the autophagic compartments in dying cells^11^,^12^,^13^,^14^,^15^,^16^, attention was directed to the signals that are involved in triggering autophagy in these tissues^17^,^18^,^19^.

In the last few years, most of the genes related to the autophagic process have been identified in the silkworm genome. These include autophagy-related (*ATG*) genes and genes coding for proteins that belong to the phosphatidylinositol 3-kinase (PI3K) signal transduction pathway^20^,^21^. More recently, significant efforts have been devoted to the characterization of genes that are involved in the autophagic process in these insects, and several studies have analysed gene expression patterns in the larval tissues of different Lepidoptera in the attempt to establish a correlation between the activation stimulus (i.e., developmental or starvation signals) and the role of autophagy in each context^8^,^9^,^21^,^22^.

A phenomenon that has been widely described in several organs that die during metamorphosis in Lepidoptera is the coexistence of autophagic and apoptotic features^3^,^5^,^7^,^8^,^9^,^23^,^24^,^25^,^26^,^27^. This evidence has prompted the hypothesis of a causative role of autophagy in the demise of these tissues, although the existence of autophagic cell death in these insects has never been clearly and definitively demonstrated^21^,^28^.

Indeed, the role of autophagy as a pro-death or pro-survival process in insect development is puzzling, and it is believed to be context-dependent. For example, in *Drosophila melanogaster*, which represents a reference model among insects, autophagy was demonstrated to be required for the removal of salivary glands and the midgut^29^,^30^. However, apoptosis, and not autophagy, was shown to be the major force driving abdominal muscle remodelling^31^ and to be necessary during spermatid differentiation^32^. In Lepidoptera, the role of this process during development still needs to be assessed, and only limited information is available. For example, Tian *et al*.^9^ suggested that autophagy promotes lipolysis in the fat body, while studies performed on the midgut hypothesized that autophagy provides trophic support to the epithelium during metamorphosis^5^,^7^.

An important aspect that is worthy of consideration when studying the remodelling or removal of larval tissues is hormonal regulation. In fact, it has been proven that hormones play a major role in regulating programmed cell death (PCD)-related processes in both *Drosophila* and Lepidoptera. In particular, 20-hydroxyecdysone (20E) is the master regulator of insect metamorphosis and can activate both autophagy and apoptosis during insect development^9^,^26^,^33^. However, the regulation of autophagy and apoptosis by hormonal cues in the same lepidopteran organ has never been studied. This topic appears to be worthy of investigation if we consider that at least two peaks in ecdysone titres occur within the haemolymph during the larva-pupa transition when autophagy and/or apoptosis are being activated in various larval organs^34^.

In this study, we characterized in detail the process of degeneration in the silkworm midgut during metamorphosis. Whereas certain organs, such as fat body and anterior silk glands, are only remodelled during the larva-pupa transition and still persist in the adult insect, the larval midgut is completely degraded. For this reason, this organ represents an interesting tissue for studying cell death under hormonal stimulation during development and for evaluating the roles of both autophagy and apoptosis, as well as their relationship. During the larva-pupa transition, the larval midgut epithelium of lepidopterans is progressively displaced by a new epithelial layer that grows underneath it; the larval midgut is pushed into the gut lumen and forms the yellow body, a compact mass of cells that subsequently die. During this complex developmental process, features typical of both apoptosis and autophagy were reported in degenerating cells^5^,^7^. In a previous study, we showed that features of autophagy and apoptosis occur at different times in this setting^7^, but several aspects, such as the regulation and role of the two processes in this scenario, were not demonstrated. To fill this gap of knowledge, we have now analysed the autophagic and apoptotic processes both at the molecular and functional level. Moreover, we investigated how 20E regulates these processes and we used chemical modulators of both apoptosis and autophagy to understand the role of the two processes in this scenario. To our knowledge, this represents the first comprehensive study of autophagy and apoptosis within the same organ and the first attempt to unravel the complexity of their regulation.

## Results

### Autophagy is activated at the beginning of metamorphosis

**Figure 1:**
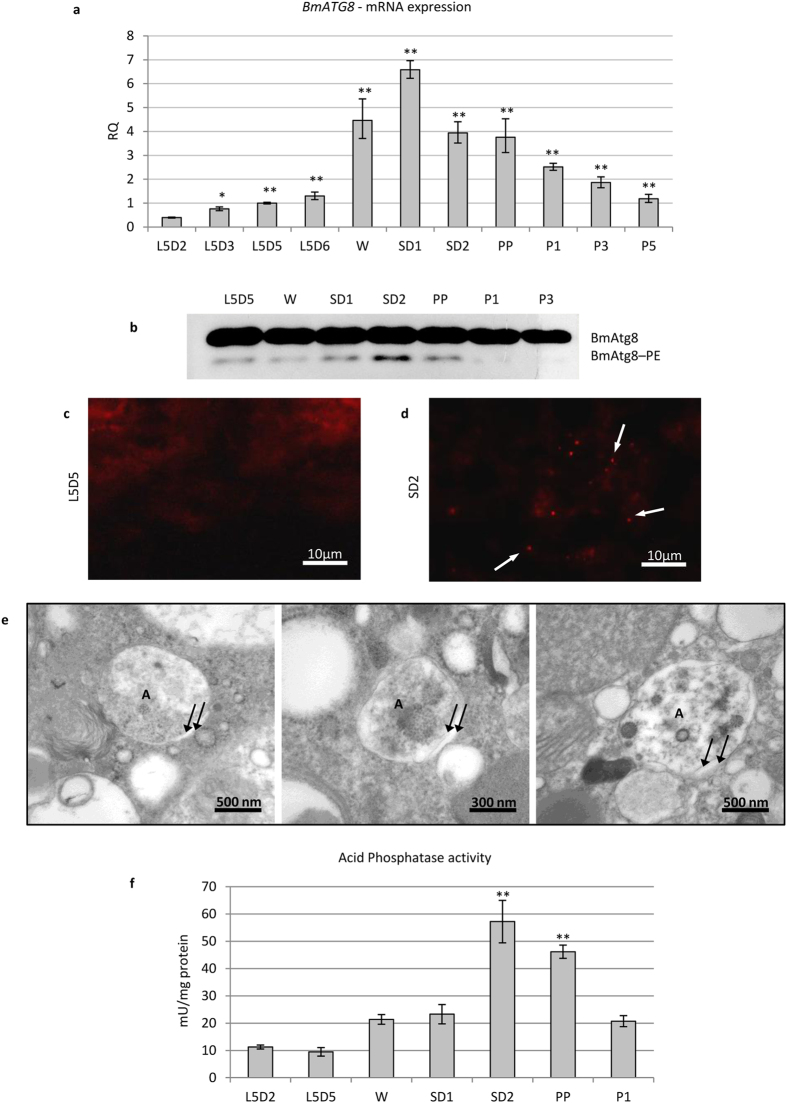
Autophagy is activated at spinning stage. (**a**) qRT-PCR analysis of *BmATG8* mRNA levels in midgut tissues; (**b**) Western blot analysis of BmATG8–PE; (**c,d**) immunofluorescence staining of BmAtg8 showing the presence of autophagosomes (arrows) in midgut tissues at SD2 (**d**) compared to L5D5 (**c**) stage; (**e**) TEM images of autophagosomes in midgut cells at SD2 stage (A: autophagosome; arrows: autophagosome membranes); (**f**) analysis of acid phosphatase activity in midgut cells. Values represent mean ± s.e.m. (*p < 0.05; **p < 0.01 compared to L5D2 using ANOVA followed by Tukey’s HSD test).

In previous work from our group, the presence of autophagic features in the midgut of *Bombyx mori* during metamorphosis was reported^7^. In that study, however, we did not assess the precise timing of autophagy activation. To clarify this aspect, we first analysed the expression of the gene *BmATG8* at different time points by using quantitative reverse transcription PCR (qRT-PCR), starting from the fifth larval instar up to the pupal stage. *BmATG8* mRNA levels increased at the end of the last larval instar and peaked at the SD1 stage (Fig. 1a).

To confirm autophagy activation in midgut cells, we measured BmAtg8–PE levels by using a commercially available anti-Gabarap antibody (Supplementary Fig. S1). Western blot analysis of midgut samples obtained from *B. mori* at different time points showed that BmAtg8–PE levels increased at the SD2 stage (Fig. 1b). Immunostaining performed by using the same antibody showed BmAtg8–positive puncta in the midgut epithelium during SD2, thus demonstrating the presence of autophagosomes in the midgut tissue at this stage (Fig. 1d). Conversely, only scarce BmAtg8–positive puncta were visible at L5D5 (Fig. 1c). The presence of numerous autophagosomes at SD2 was confirmed by transmission electron microscopy (TEM) (Fig. 1e), while they were scarcely represented during the larval stage (L5D5). The analysis of acid phosphatase activity showed that the lysosomal pathway was activated at SD2 (Fig. 1f). Collectively, these data demonstrate that, at the transcriptional level, the autophagic process is activated at the beginning of metamorphosis (W-SD1 stage) in silkworm midgut, while, as confirmed by the increases in the expression of BmAtg8–PE and acid phosphatase activity, the maximum occurrence of autophagy takes place later (SD2).

### Activation of apoptosis follows autophagy

**Figure 2:**
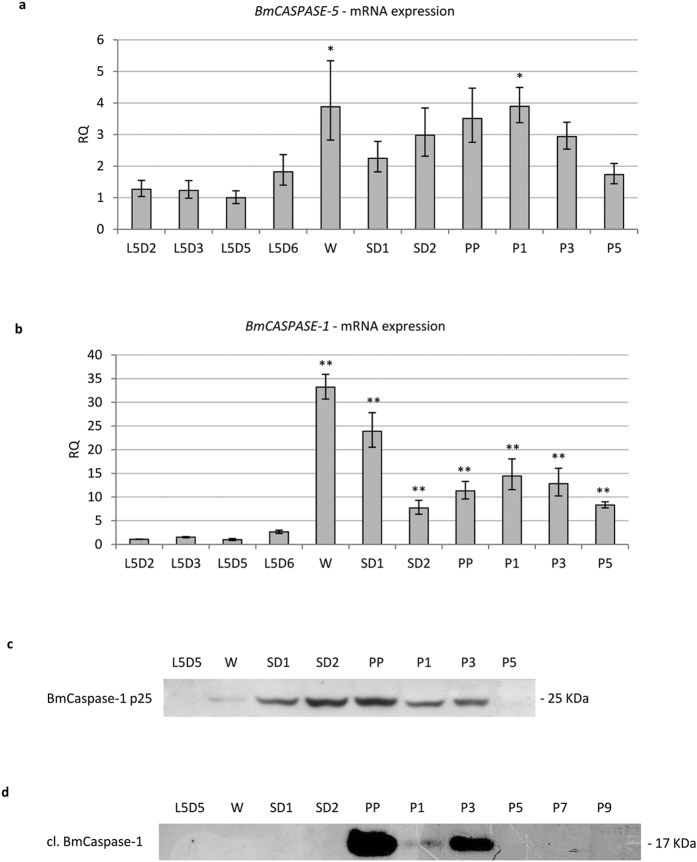
Apoptosis is activated after autophagy. (**a,b**) qRT-PCR analysis of *BmCASPASE-5* (**a**) and *BmCASPASE-1* (**b**); (**c**,**d**) Western blot analysis of uncleaved (**c**) and cleaved (**d**) BmCaspase-1 during metamorphosis. Values represent mean ± s.e.m. (*p < 0.05; **p < 0.01 compared to L5D2 using ANOVA followed by Tukey’s HSD test).

Franzetti *et al*.^7^ reported the co-existence of autophagic and apoptotic features during degeneration of the larval midgut in *B. mori*. Here, we monitored two caspases to analyse the time course of apoptosis. In particular, we evaluated the expression of *BmCASPASE-5*, a gene that codes for an initiator caspase, and *BmCASPASE-1*, which codes for an effector caspase. BmCaspase-5 is the lepidopteran homologue of *Drosophila* Dronc^35^, a key initiator caspase involved in developmental apoptosis whose mRNA is highly transcribed during metamorphosis^36^,^37^. BmCaspase-1 is the homologue of *Drosophila* Drice and Dcp-1 and is considered the main effector caspase in Lepidoptera^35^. The expression pattern of *BmCASPASE-5* showed two peaks at the W and P1 stages (Fig. 2a). *BmCASPASE-1* gene expression resembled that of *BmCASPASE-5* because it was strikingly upregulated during W-SD1 and reached a second, lower peak during the first day of the pupal stage (Fig. 2b).

Given that the production of active Caspase-1 requires a two-step cleavage of the zymogen (procaspase)^38^, we evaluated the expression levels of both uncleaved and cleaved BmCaspase-1 proteins in midgut cells. The anti-cleaved Caspase-3 antibody used in this work was described to detect caspases in *Drosophila*^39^,^40^,^41^. Moreover, it must be underscored that, among *B. mori* caspases, the epitope ETD, which is recognized by this antibody^40^, is present only in the BmCaspase-1 sequence. In silkworm midgut, the anti-Caspase-3 antibody detected a 25-kDa band whose molecular weight was comparable to the inactive p25 Caspase-1 intermediate (Fig. 2c)^38^,^42^, whereas the anti-cleaved Caspase-3 antibody recognized a band of 17-kDa, the expected molecular weight of cleaved Caspase-1 (Fig. 2d)^38^,^42^. Uncleaved BmCaspase-1 (p25) could be detected from the W stage to the P3 stage (Fig. 2c). However, no expression of cleaved BmCaspase-1 could be observed in midgut tissues until SD2: the activated form of the effector caspase was highly expressed at the PP stage and, although at lower levels, it was present until P3 (Fig. 2d).

These data collectively show that the transcriptional induction of apoptotic machinery occurs at the W-SD1 stage. Starting from the W stage, the presence of uncleaved BmCaspase-1 can be detected in the midgut tissues. However, activation of the effector caspase is observed only from the PP stage.

### Tor inhibition is not sufficient to mediate the activation of autophagy by 20E

In the haemolymph of Lepidoptera, the 20E titre has two peaks during the larva-pupa transition: the commitment peak at the wandering stage and the metamorphic peak during the pupal stage^43^. Although we observed that autophagic gene transcription is triggered concurrently with the commitment peak^34^,^44^ (Fig. 1a), a causal relationship between the two events in the midgut must be proven. We therefore analysed the mechanism underlying autophagy induction by ecdysone in the midgut epithelium by injecting fifth instar larvae (L5D2 stage) with 20E (10 μg/larva).

**Figure 3:**
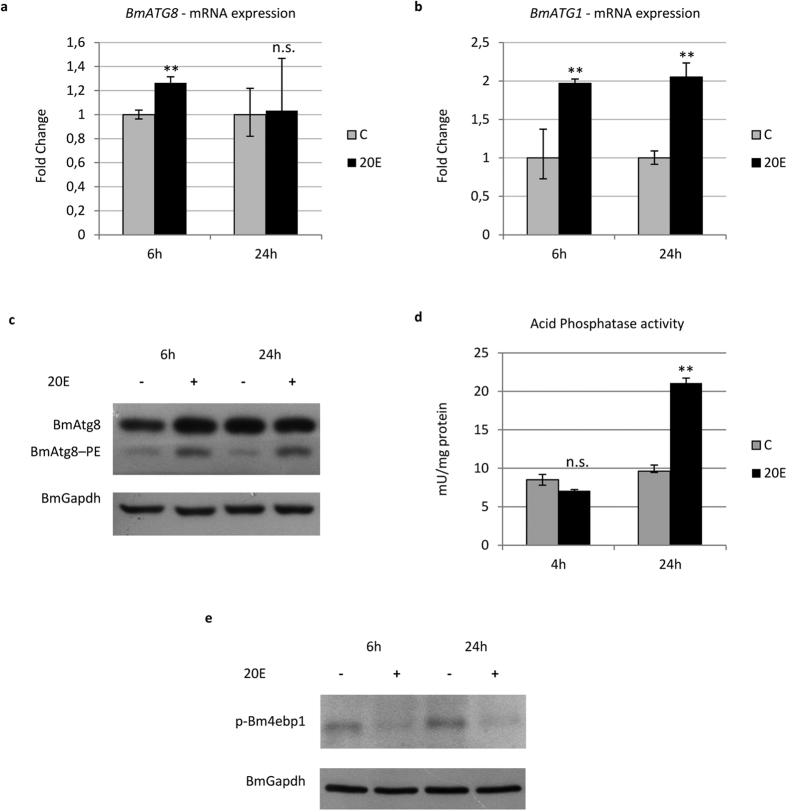
Autophagy is activated by 20E. (**a**,**b**) qRT-PCR analysis of *BmATG8* (**a**) and *BmATG1* (**b**) in midgut cells after administration of 20E; (**c**,**d**) Western blot analysis of BmAtg8–PE (**c**) and acid phosphatase activity (**d**) in midgut of larvae injected with the hormone; (**e**) Western blot analysis of p-Bm4ebp1 in hormone-treated larvae. Values represent mean ± s.e.m. (**p < 0.01 using Student’s *t*-test).

qRT-PCR analysis detected overexpression of the *BmATG8* (Fig. 3a) and *BmATG1* (Fig. 3b) genes following 20E administration, and a strong increase in BmAtg8–PE was observed after the hormone treatment (Fig. 3c). The rise in acid phosphatase activity confirmed full induction of the autophagic process (Fig. 3d). It was previously demonstrated that 20E inhibits the protein Tor (target of rapamycin) both in *Drosophila* and *B. mori* fat body, thus eliciting the autophagic process^9^,^33^. However, previous studies have also reported that 20E activates autophagy directly, affecting the expression of specific *ATG* genes (such as *ATG1*), and thus independently of inhibition of the Tor pathway^9^.

**Figure 4:**
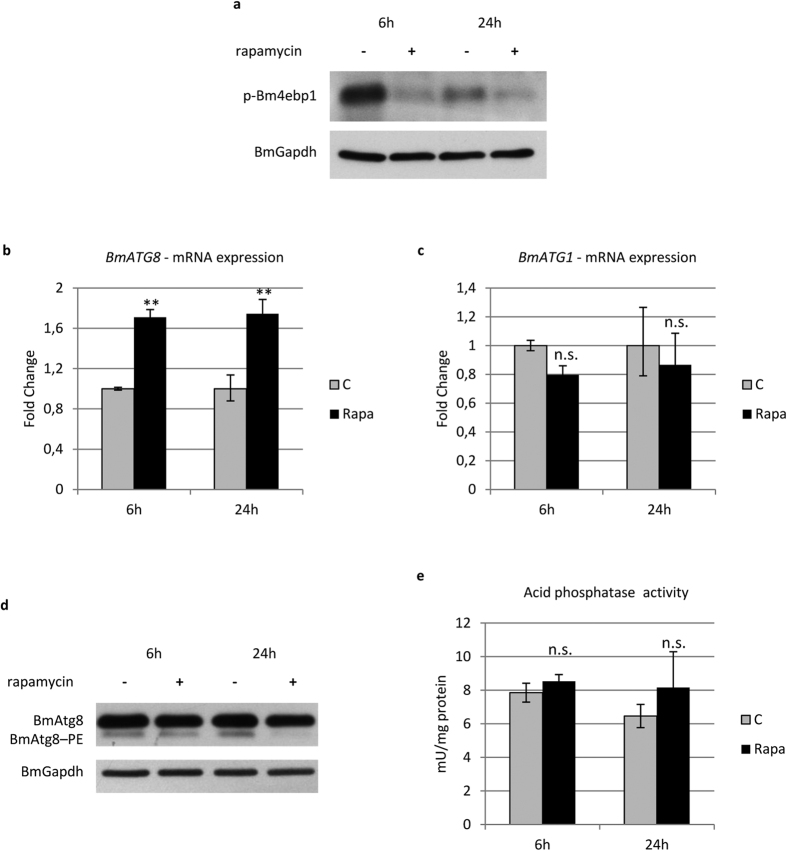
Rapamycin fails to activate a full autophagic response. (**a**) Western blot analysis of p-Bm4ebp1 in midgut of larvae injected with rapamycin; (**b**,**c**) qRT-PCR analysis of *BmATG8* (**b**) and *BmATG1* (**c**) expression after rapamycin treatment; (**d,e**) Western blot analysis of BmAtg8–PE (**d**) and acid phosphatase activity (**e**) after administration of rapamycin. Values represent mean ± s.e.m. (**p < 0.01 using Student’s *t*-test).

To verify the involvement of Torc1 (Tor complex 1) in 20E-induced autophagy in midgut cells, we evaluated the activity of this complex by monitoring the phosphorylation levels of its target, 4ebp1. Western blot analyses showed that Torc1 activity substantially decreased after the treatment (Fig. 3e). To verify whether 20E activates autophagy by acting exclusively on Torc1, we evaluated the effects of inhibiting this complex on autophagy induction by treating larvae with rapamycin. Following rapamycin administration, the levels of phosphorylated 4ebp1 showed a strong decrease, similar to that observed after 20E injection (Fig. 4a). Indeed, the inhibition of Torc1 during the last larval instar increased the expression of *BmATG8* at 6 and 24 h after rapamycin was administered (Fig. 4b). However, rapamycin was not able to induce *BmATG1* expression (Fig. 4c). Surprisingly, BmAtg8–PE levels decreased following treatment with rapamycin (Fig. 4d), while acid phosphatase activity did not increase significantly after the injection (Fig. 4e), two results confirming the occurrence of an incomplete autophagic response in rapamycin-treated larvae.

From these data we can conclude that 20E can upregulate *ATG* genes and activate autophagy. Moreover, we demonstrated that 20E acts on Torc1 to induce autophagy, although activation of this complex alone is not sufficient to activate and sustain a full autophagic response in midgut cells.

### Apoptosis is finely regulated by 20E titre

**Figure 5:**
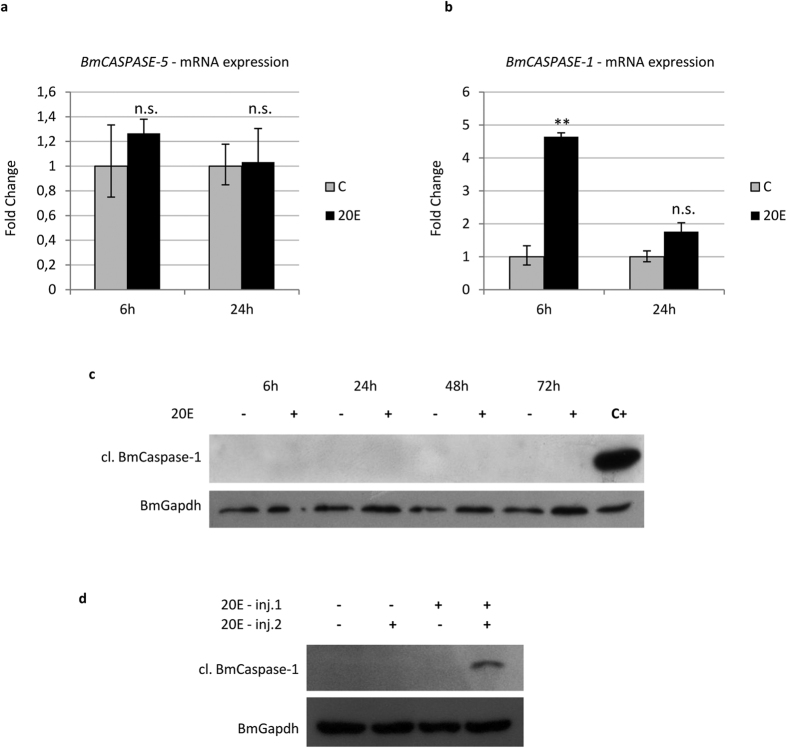
Apoptosis is activated by 20E. (**a,b**) qRT-PCR analysis of *BmCASPASE-5* (**a**) and *BmCASPASE-1* (**b**) in midgut of larvae injected with 20E; (**c,d**) Western blot analysis of cleaved BmCaspase-1 after single (**c**) or double (**d**) administration of 20E. Values represent mean ± s.e.m. (**p < 0.01 using Student’s *t*-test).

Similar to *ATG* genes, caspase mRNA levels are upregulated concurrently with 20E commitment peak^34^,^44^ (Fig. 2a,b). To study the effects of 20E on the induction of apoptosis in midgut cells, we planned a set of experiments based on injection of this hormone during the last larval instar. No appreciable upregulation of the initiator caspase gene *BmCASPASE-5* was observed after injecting a single dose of 20E (10 μg/larva) (Fig. 5a), while a significant increase in *BmCASPASE-1* mRNA levels was recorded 6 h later (Fig. 5b). However, a single dose of 20E was not able to activate the BmCaspase-1 protein up to 72 h following the injection (Fig. 5c).

These data suggest that, under physiological conditions, the 20E commitment peak can upregulate *BmCASPASE-1* expression, but it cannot trigger BmCaspase-1 activation. To confirm the hypothesis that apoptosis is induced by the 20E metamorphic peak, we designed an injection protocol that mimicked the physiological pattern of ecdysone levels occurring in the larva during metamorphosis. For this purpose, we administered 20E (50 μg/larva) a second time 24 h after the first hormone injection (10 μg/larva). As a result of the double administration, BmCaspase-1 activation was observed in 30% of the larvae 48 h after the second injection. In contrast, no caspase activation was observed in any of the larvae treated with a single dose of 20E (10 μg or 50 μg 20E/larva) (Fig. 5d). Our data confirm that, during metamorphosis, the first rise in the 20E titre within the haemolymph (commitment peak) is needed to upregulate the expression of apoptotic genes, while caspase activation is triggered only after the occurrence of the second 20E peak (metamorphic peak).

### Autophagy has a pro-survival role in midgut cells undergoing degeneration

As demonstrated above, in the silkworm midgut, autophagy precedes apoptosis, but its role as a pro-survival or pro-death mechanism is still a matter of debate. To gain insight into how this process functions in midgut cells undergoing degeneration, we used RNA interference (RNAi) to silence *ATG* genes. Unfortunately, this genetic approach failed and did not generate significant outcomes in our model (Supplementary Fig. S2), and therefore, we decided to inhibit autophagy by using chloroquine and wortmannin. Chloroquine is a widely used autophagy inhibitor that acts on the lysosome-autophagosome fusion step, thus blocking the autophagic flux, while wortmannin acts on class III PI3K proteins upstream of autophagy activation. Larvae were injected with chloroquine or wortmannin at the beginning of autophagy activation (stage SD1).

**Figure 6:**
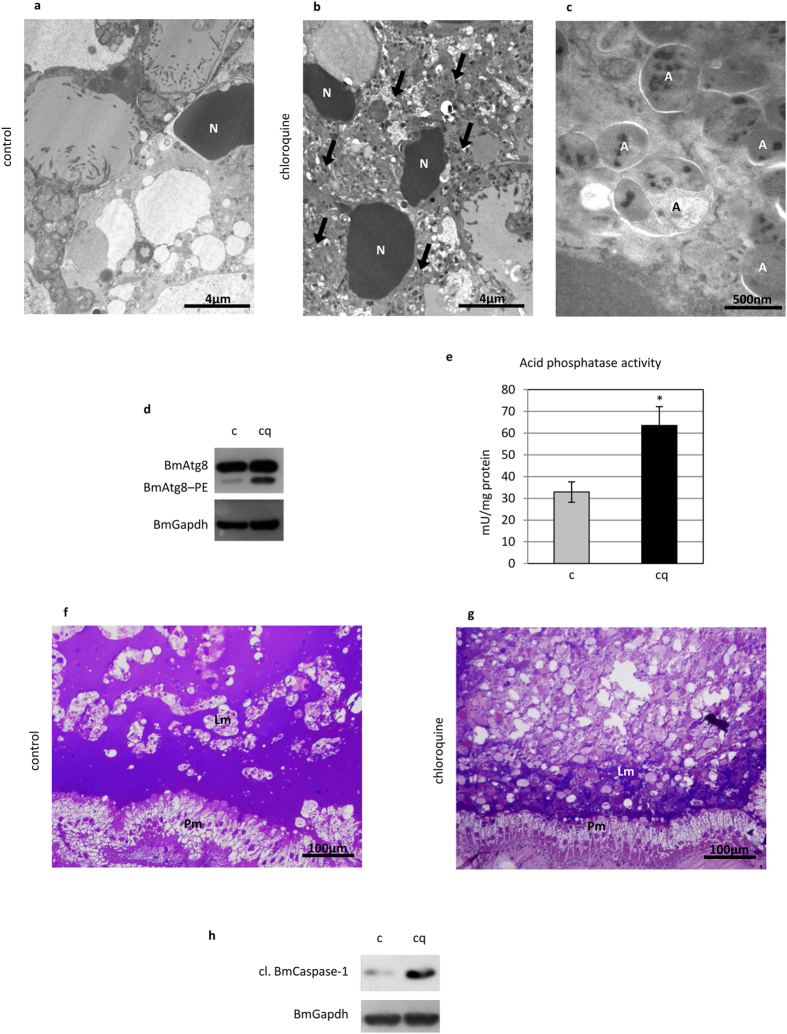
Autophagy inhibition by chloroquine determines an increased degeneration of the larval midgut. (**a,b**) TEM analysis of midgut cells in control (**a**) and chloroquine-treated (**b**) insects showing a significant accumulation of vesicles (arrows) after inhibition of autophagy; (**c**) detail of autophagic compartments in midgut cells of treated larvae; (**d,e**) Western blot analysis of BmAtg8–PE (**d**) and acid phosphatase activity (**e**) demonstrating the inhibition of autophagy in midgut cells by chloroquine; (**f,g**) morphology of the larval and pupal epithelium in control (**f**) and treated (**g**) pupae; (**h**) Western blot analysis of cleaved BmCaspase-1 in midgut cells of larvae treated with chloroquine. A: autophagosomes; Lm: larval midgut epithelium; N: nucleus; Pm: pupal midgut epithelium. Values represent mean ± s.e.m. (*p < 0.05 using Student’s *t*-test).

TEM analysis showed that many vesicles accumulated in midgut cells after administering chloroquine (Fig. 6a–c). According to their size, morphology, and content, they could be classified as autophagic compartments. Moreover, Atg8–PE levels (Fig. 6d) and acid phosphatase activity both (Fig. 6e) increased in treated larvae. These results demonstrate the efficacy of chloroquine treatment in blocking autophagic flux and the consequent accumulation of autophagosomes and lysosomes in midgut cells. While the morphology of the new pupal epithelium did not change in larvae injected with chloroquine, the organization of the larval epithelium was completely different. In fact, the old epithelium appeared more degenerated than in control animals, and no tissue structure was visible after the treatment (Fig. 6f,g). Furthermore, Western blot analysis showed increased levels of cleaved BmCaspase-1 after administration of the autophagic inhibitor (Fig. 6h).

**Figure 7:**
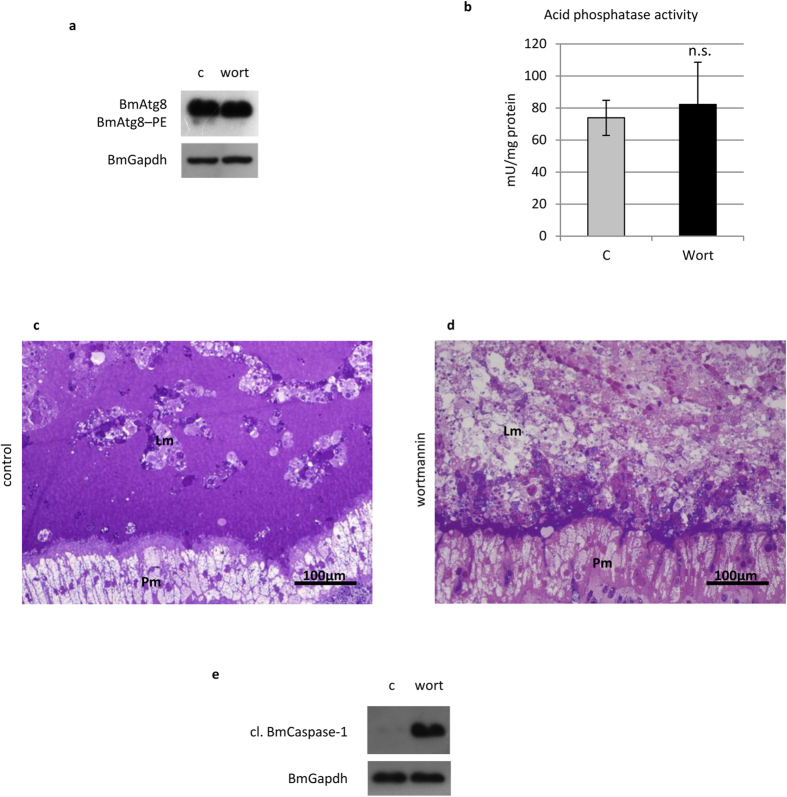
Wortmannin impairs autophagy and leads to increased degeneration of the midgut epithelium. (**a**) Western blot analysis of BmAtg8–PE demonstrating autophagy inhibition in midgut cells treated with wortmannin; (**b**) acid phosphatase activity; (**c,d**) morphology of larval and pupal epithelium in control (**c**) and treated (**d**) pupae; (**e**) Western blot analysis of cleaved BmCaspase-1 in midgut cells of larvae treated with wortmannin. Lm: larval midgut epithelium; Pm: pupal midgut epithelium. Values represent mean ± s.e.m.

Lower Atg8–PE levels were visible in the larvae treated with wortmannin (Fig. 7a) due to the impairment of autophagy activation. Acid phosphatase activity did not show any significant change (Fig. 7b), indicating that, conversely to chloroquine treatment, lysosomes had not accumulated in the midgut cells. The effects of the inhibitor on the larval midgut were comparable to those obtained by chloroquine (Fig. 7c,d), and an increase in cleaved BmCaspase-1 levels could also be observed (Fig. 7e). Of note, although the two inhibitors act at different levels of the autophagic process (autophagosome-lysosome fusion vs. an upstream step of autophagy activation, i.e., class III PI3K activity), a similar morphological effect on the tissue was observed after the treatments. Moreover, increased levels of active BmCaspase-1 were recorded after inhibiting autophagy in both cases. These data demonstrate that inhibition of autophagy determines a stronger degree of degeneration of the midgut epithelium and increased levels of activated caspases in these cells, thus suggesting a pro-survival role of autophagy in this tissue.

### Apoptosis drives the demise of midgut cells

**Figure 8:**
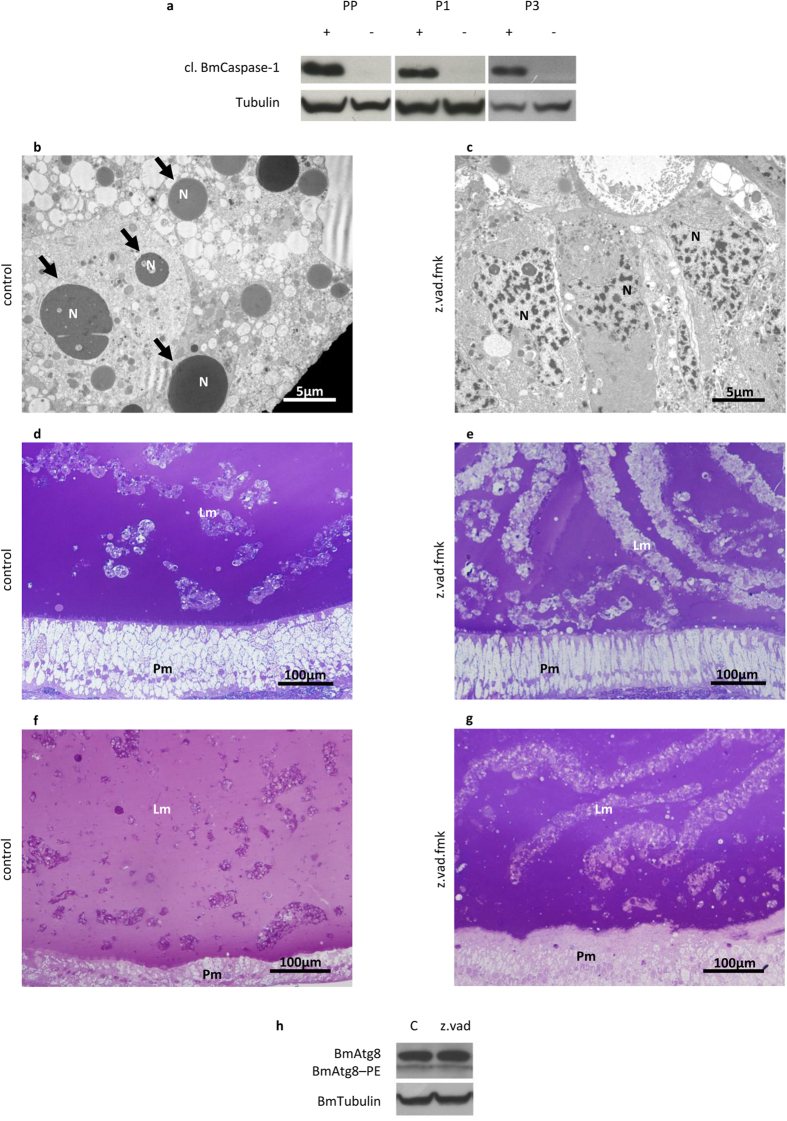
Caspase inhibition delays the degeneration of the midgut epithelium. (**a**) Western blot analysis of cleaved BmCaspase-1 in midgut of larvae treated with z.vad.fmk. The absence of activated caspase expression can be appreciated at PP, P1, and P3 stages following the administration of the inhibitor at SD2 stage; (**b,c**) TEM analysis of midgut in control (**b**) and treated (**c**) larvae at PP stage; (**d,e**) morphology of larval and pupal epithelium in control (**d**) and treated (**e**) P3 pupae; (**f,g**) morphology of larval and pupal epithelium in control (**f**) and treated (**g**) P9 pupae; (**h**) Western blot analysis of BmAtg8–PE in the midgut of z.vad.fmk-treated larvae. Lm: larval midgut epithelium; N: nucleus; Pm: pupal midgut epithelium; arrows: apoptotic nuclei.

Generally, apoptosis is considered the key player in tissue degeneration during development. However, caspases do not always need to be activated for removal of larval organs during insect metamorphosis^29^. To unravel this aspect, we targeted caspase activation by using z.vad.fmk, which is able to inhibit both initiator and effector caspases, thus blocking the apoptotic process. We used the lowest concentration of inhibitor able to completely suppress the activation of BmCaspase-1. Moreover, we verified that the inhibitor was fully effective only if injected during the SD2 stage, just before BmCaspase-1 activation (Supplementary Fig. S3). Larvae treated with the inhibitor showed undetectable levels of cleaved BmCaspase-1 24, 48 and even 96 h after the treatment (the PP, P1, and P3 stages, respectively) (Fig. 8a). TEM analysis revealed the presence of a limited number of apoptotic nuclei in these larvae compared to controls (Fig. 8b,c). A significant alteration in the morphology of the larval epithelium was visible in treated animals. In fact, while in control P3 pupae, the epithelium was clearly degenerated (Fig. 8d), an epithelial-like morphology could still be observed in treated animals (Fig. 8e). The effect of caspase inhibition on the larval epithelium was even more evident at the P9 stage (Fig. 8f,g). No alteration in the autophagic flux was observed after z.vad.fmk treatment, thus excluding an interaction between caspase activation and autophagy (Fig. 8h). Our experiments collectively demonstrate that apoptosis is sufficient to drive larval midgut degeneration during silkworm metamorphosis.

## Discussion

Previous studies have described the occurrence of autophagy and apoptosis in the larval organs of Lepidoptera^5^,^7^,^8^,^9^,^21^,^26^,^27^. However, autophagy and apoptosis show peculiar features in the tissues and organs of insects, and their roles seem to be context-dependent. For this reason, many aspects still remain unclear. With the aim to fill this gap of knowledge, we analysed the timing of the occurrence of autophagy and apoptosis during the demise of silkworm midgut. Moreover, we performed functional experiments by using activators and inhibitors to gain insight into their regulatory mechanisms and roles in midgut remodelling during metamorphosis. To our knowledge, this is the first study to investigate in detail both autophagy and apoptosis in an *in vivo* lepidopteran model.

The appropriate criteria for assessing the activation of autophagy are often a matter of debate, especially when unconventional model systems are studied^45^. To evaluate autophagy activation and to follow this process during metamorphosis, we tested different direct and indirect autophagic markers in *B. mori* midgut.

We demonstrated that the mRNA levels of *BmATG8*, a key protein in autophagic machinery, increase in this organ at the beginning of metamorphosis. Moreover, we showed that, following the transcriptional upregulation of this gene, the autophagic process is activated. Thus, although Klionsky *et al*.^45^ reported that assessing the mRNA levels of autophagy-related genes may provide only correlative data related to the induction of autophagy, our results show that the increased expression of *ATG* genes can be considered an indirect marker of this process, at least in our model. In addition, our data clearly confirm that autophagy occurs in the developmental remodelling of *B. mori* midgut during metamorphosis, as has also been shown for *H. virescens*^5^ and *Drosophila*^29^ midguts, as well as for other silkworm organs^6^,^9^,^27^.

We showed that the apoptotic process is regulated differently than autophagy. In particular, the analysis of caspase mRNA levels demonstrates that apoptosis is activated at the transcriptional level concomitantly with the upregulation of *ATG* genes. The inactive form of the effector Caspase-1 is stored in the cytoplasm at the beginning of metamorphosis, but it is not activated until the prepupal stage. A subsequent but lower activation of apoptosis at the gene (P1) and functional (P3) level is observed at the beginning of the pupal stage. We hypothesize that the presence of two rounds of caspase activation might be necessary for a two-step process in the demise of different midgut regions or cell types (e.g., columnar vs goblet cells). This hypothesis is corroborated by a previous study of the midgut of *Galleria mellonella*, where three different apoptotic waves were required for degeneration of distinct parts of the midgut epithelium^25^.

20E is usually regarded as a regulator of autophagy in various insect models. Similarly to what occurs in *Drosophila* fat body^33^, it was shown that autophagy can be stimulated by 20E in the fat body of *B. mori*: this hormone activates autophagy by inhibiting the Tor pathway, thus allowing for activation of the downstream Atg1 complex^9^.

Our results demonstrate that 20E can trigger autophagy in the midgut of *B. mori* during metamorphosis. In particular, activation of autophagy is mediated by the first rise in 20E concentration in the haemolymph (20E commitment peak). We also demonstrate that Torc1 is involved in the molecular response to 20E, similarly to what occurs in *Drosophila*^33^ and *B. mori*^9^ fat body. Nonetheless, Torc1 inhibition alone is not sufficient to activate a full autophagic response, suggesting that 20E acts concurrently on more than one target. Accordingly, unlike 20E, rapamycin fails to stimulate the expression of *BmATG1*. This result is consistent with the presence of an ecdysone response element in the promoter of this gene^9^, which likely permits transcriptional regulation by 20E without involving the Tor pathway. For these reasons, we hypothesize that Torc1 is involved in activating autophagy, but it cannot induce the production of all the components needed to elicit a full autophagic response. Thus, following rapamycin treatment, autophagic flux is activated, and BmAtg8–PE, which is already localized on autophagosomes, is degraded. In this setting, because no *de novo* synthesis of autophagosomes occurs, the final outcome is an overall decrease in BmAtg8–PE levels. In accordance with our results, Tian *et al*.^9^ showed that autophagy could be moderately induced by injecting rapamycin in feeding larvae, but the effects elicited were weaker than those obtained by administering 20E.

Because the temporal pattern of the apoptotic process differed from that of autophagy, we designed a set of experiments to investigate the role of 20E in regulating apoptosis. Our results show that a single dose of 20E is able to upregulate *BmCASPASE-1*, confirming a role for this hormone in the transcriptional regulation of apoptotic factors. In contrast, because no upregulation of *BmCASPASE-5* was observed after the hormonal treatment, we hypothesized that the transcription of this initiator caspase might be regulated *in vivo* by a different stimulus or that other factors are needed for 20E to affect this gene. The administration of a single dose of 20E cannot trigger apoptosis, while two subsequent doses of 20E, which mimic the commitment and metamorphic peak, need to be injected to induce the cleavage and activation of BmCaspase-1. Taken together, our data demonstrate that the first 20E pulse, which occurs during the larva-pupa transition, activates the transcriptional regulation of apoptotic genes and the translation of effector caspases; thereafter, the 20E metamorphic peak is necessary to stimulate activation. A similar model was proposed by Manaboon *et al*.^43^ for the degeneration of anterior silk glands during metamorphosis.

In *Drosophila*, steroid-induced apoptosis is controlled by a balance between inhibitor of apoptosis protein (diap1), which prevents the activation of caspases, and its repressors reaper and hid^46^. We have already shown that, in the silkworm, the inhibitor of apoptosis protein *BmIAP* is highly expressed at the end of the last larval stage^7^. Moreover, to obtain insight into the mechanisms that trigger apoptosis, we evaluated mRNA expression of the inhibitor of apoptosis protein *BmIAP2* and of the pro-apoptotic protein *BmIBM1*, the homologue of *Drosophila* reaper^47^. Our gene expression data indicate that anti-apoptotic proteins are highly expressed in the midgut when the 20E commitment peak occurs (Supplementary Fig. S4). This event can reasonably block the activation of caspases. Conversely, after the spinning stage and concomitantly with the occurrence of the 20E metamorphic peak, the expression of both *BmIAP* and *BmIAP2* decreases, while *BmIBM1* mRNA levels reach a maximum (Supplementary Fig. S4), a clear indication of the shift of the cells to the apoptotic condition. According to this preliminary evidence, we hypothesized that the two-step process needed to activate apoptosis following the 20E peaks may depend on the differential expression of key apoptotic regulators.

In *Drosophila,* both the regulation and the roles of autophagy and apoptosis have been reported to be context-specific. For example, cell death in the abdominal muscles occurs by apoptosis and does not require autophagy^31^. Apoptosis was also initially regarded as the major force driving the demise of the midgut^46^, although a subsequent study noted that autophagy is the key process needed for degeneration of this organ, whereas caspase activity appears to be dispensable^29^. Autophagy is also required for activating caspase-dependent cell death for removal of the salivary gland and during oocyte maturation^30^,^48^. Although the functions of autophagy and apoptosis were hypothesized to be tissue-specific in Lepidoptera^7^,^8^,^9^,^25^,^26^, functional studies aiming to unravel their roles have never been performed. Our data show that the midgut epithelium of larvae treated with the autophagy inhibitors chloroquine and wortmannin present a more degraded morphology and enhanced levels of caspases. These results demonstrate that autophagy is not involved in the cell death of the *B. mori* larval midgut during metamorphosis, in contrast to that of the *Drosophila* midgut^29^.

These data corroborate the evidence obtained in previous studies by our group^7^,^49^ and suggest a dual role for autophagy in the midgut: i) it permits larval midgut cells to survive starvation during early metamorphosis, and ii) it allows for the catabolic degradation of the cell components that can be used later by the newly forming pupal-adult epithelium. In fact, Franzetti *et al*.^7^,^49^ showed that autophagy is responsible for the production of high ATP levels in the midgut during metamorphosis^7^ and that the new midgut epithelium can recycle and absorb molecules derived from the degeneration of larval midgut cells^49^. Moreover, an involvement of autophagy in the catabolic response triggered by starvation was hypothesized in the fat body, where energy must be produced during metamorphosis^9^. Accordingly, we have shown that, if autophagy is inactivated, a higher degree of degeneration of the midgut tissue is observed. We speculated that, following autophagy inhibition, cells cannot counteract the lack of nutrients and, as a consequence, caspase activation is enhanced, thus triggering alternative death mechanisms.

Our results confirm that apoptosis is the major process responsible for the degeneration of the larval midgut during metamorphosis. Accordingly, inhibition of caspases strongly delays the demise of the epithelium. In addition, the evidence provided herein seems to exclude the occurrence of autophagic cell death in this tissue.

In summary, we demonstrated that under physiological conditions, the first rise in 20E stimulates the transcription of both autophagic and apoptotic genes. Autophagy is activated soon thereafter, likely enabling cells to survive nutrient deprivation and helping to recycle cell components. However, apoptosis is switched on later by the metamorphic rise in 20E and leads to the demise of the midgut cells. The cytoplasmic content, released from these decaying cells in the lumen, can be absorbed by the new pupal epithelium, as previously demonstrated^49^.

## Methods

### Experimental animals

Larvae of a *B. mori* four-way polyhybrid strain (126 × 57)(70 × 90) were provided by the CREA - Honey Bee and Silkworm Research Unit (Padova, Italy). The larvae were fed on artificial diet^50^ and reared at 25 ± 0.5 °C under a 12:12-h light:dark period and at 70% relative humidity. After animals had ecdysed to the last larval instar, they were staged and synchronized according to the morphological features listed in Supplementary Table S5. Silkworms at the desired stage were quickly anesthetized with CO_2_. Midgut dissection was performed by using a SZ30 stereomicroscope (Olympus, Tokyo, Japan).

### Functional experiments

To analyse the roles and regulation of autophagy and apoptosis, larvae were treated during the feeding or spinning stage with the following chemicals: 20E (10–50 μg/larva, Sigma, H5142), rapamycin (10 μg/larva, Sigma, R8781), z.vad.fmk (25 μg/larva, Promega, G7231), chloroquine (1 mg/larva, Sigma, C6628), and wortmannin (40 μg/larva, Sigma, W3144). For 20E experiments, preliminary assays were carried out to assess the lowest amount of hormone able to reproduce morphological features typical of larvae at the wandering stage^44^. For apoptotic and autophagic inhibitors, the chosen concentration was able to elicit an inhibitory response without any toxic effects, such as larval death and/or abnormal development of the pupa. Injections were performed in the first pair of prolegs to avoid damaging internal organs by using a 10-μl syringe (Hamilton, 701N). Control larvae were injected with an equal amount of solvent. Larvae were collected 6, 24, 48, and/or 72 h after the treatment for morphological and molecular analyses (see the “Results” section for further information).

### qRT-PCR

Midguts were dissected on ice, cleansed of fat body and tracheae residues, and immediately frozen in liquid nitrogen until use. Total RNA was isolated from 20–40 mg of frozen tissue using TRIzol reagent (Life Technologies, 15596026) according to the manufacturer’s instructions. RNA was treated with a TURBO DNA-free Kit (Life technologies, AM1907) to remove possible genomic DNA contamination, and its integrity was assessed by electrophoresis. RNA was retrotranscribed to cDNA using M-MLV reverse transcriptase (Life Technologies, 28025013). The primers used for qRT-PCR are listed in Supplementary Table S6. qRT-PCR was performed using iTaq Universal SYBR Green Supermix (Bio-Rad, 1725121) and a 96-well CFX Connect Real-Time PCR Detection System (Bio-Rad, Hercules, CA, USA). The 2^−ΔΔCt^ method, with *BmRP49* as a housekeeping gene, was used to calculate the relative expression of the genes of interest. The expression of *BmRP49* was reported to be stable throughout development in *B. mori*^51^, and it has been used in previous studies on silkworm metamorphosis^9^,^26^. The efficiency of the amplification reaction for each couple of primers was adjusted within the range of 90–105%. Each value was the result of experiments performed on midguts isolated from 3 to 8 animals. Statistical analyses were performed using Student’s *t*-test or ANOVA followed by Tukey’s HSD test.

### Validation of anti-Gabarap antibody

BmAtg8 recombinant protein was expressed in BL21DE3/pET28+BmAtg8 bacteria. The pET28+BmAtg8 expression vector^52^ was provided by Prof. Yang Cao (South China Agricultural University). The anti-HsGabarap antibody (Abcam, AB109364) was tested against BmAtg8 recombinant protein and protein extracts from silkworm midgut by immunoblotting.

### Western blot analysis

Midguts were dissected and stored as described above (“qRT-PCR” section). Tissue samples (20–60 mg) were homogenized with a T10 basic ULTRA-TURRAX (IKA, Staufen, Germany) in 1 ml/0.1 g tissue of RIPA buffer (150 mM NaCl, 2% NP-40, 0.5% sodium deoxycholate, 0.1% SDS, and 50 mM Tris, pH 8.0), to which 1× protease inhibitor cocktail (Thermo Fisher Scientific, 78410) and phosphatase inhibitors (1 mM sodium orthovanadate, 5 mM sodium fluoride) were added. The particulate material was removed by centrifugation (15,000 × *g* for 15 min at 4 °C). Protein was mixed with 2× gel loading buffer and denatured by heating the samples at 98 °C for 5 min. SDS-PAGE was performed on a 12% Tris-glycine or 10% Tris-tricine acrylamide gel by loading 40 μg protein per lane. After electrophoresis, the proteins were transferred to 0.45-μm nitrocellulose (Thermo Fisher Scientific, 88018) or 0.45-μm PVDF membranes (Merck Millipore, IPVH00010). Membranes were saturated with a solution of 5% milk for 1 h at room temperature and subsequently incubated for 2 h at room temperature with the following primary antibodies: anti-Gabarap diluted to 1:2,500, anti-p4ebp1 (Cell Signalling, 236B4) diluted to 1:1,000, anti-Caspase-3 (Cell Signalling, 9662) diluted to 1:1,000, and anti-cleaved Caspase-3 (Cell Signalling, 9661) diluted to 1:1,000. Antigens were revealed with an appropriate HRP-conjugated secondary antibody anti-rabbit (Jackson Immuno Research Laboratory, 111036003) diluted to 1:7,500 or anti-mouse (Jackson Immuno Research Laboratory, 715035150) diluted to 1:5,000. Immunoreactivity was detected using LiteAblot Plus ECL (Euroclone, EMP011005). Anti-Gapdh (Proteintech, 10494-1-AP) diluted to 1:2,500 or anti-Tubulin (Sigma, T6074) diluted to 1:10,000 antibodies were used to detect housekeeping proteins. Because the use of “housekeeping proteins” as internal standards are unsuitable for comparing silkworms at different developmental stages^7^, we also adopted a Coomassie staining procedure to assess equal gel loading and blotting efficiency^53^.

### Immunofluorescence

Midguts were excised from the insects and fixed in 4% paraformaldehyde in 0.1 M phosphate-buffered saline (PBS, pH 7.2) for 3 h at room temperature. Specimens were dehydrated in an ethanol series and embedded in paraffin. Sections (8- to 10-μm-thick) were cut using a Jung Multicut 2045 microtome (Leica, Nussolch, Germany) and used for immunostaining. Sections were deparaffinized with xylene, rehydrated in an ethanol series, blocked with a solution of 2% BSA and 0.1% Tween in PBS for 30 min, and subsequently incubated with anti-Gabarap antibody (1:250) for 2 h at room temperature. After washing with PBS, sections were incubated for 1 h with a Cy5-conjugated rabbit antibody (Abcam, AB96902) diluted to 1:100. Samples were observed using an Eclipse Ni-U fluorescence microscope equipped with a DS-5M-L1 digital camera system (Nikon, Tokyo, Japan).

### Acid phosphatase activity assay

Midguts were dissected and stored, as described above (“qRT-PCR” section). After thawing, midguts were homogenized in 1 ml/0.1 g tissue in the following buffer: 100 mM mannitol and 10 mM HEPES-Tris at pH 7.2. A protease inhibitor cocktail (Thermo Fisher Scientific, 78410) was added before carrying out the homogenization procedure. The protein concentration in the homogenates was determined using the Bradford assay^54^, with BSA as a standard. Enzymatic activity in the homogenates was assayed in a reaction volume of 250 μl of 7.6 mM 4-nitrophenyl phosphate in citrate buffer (45.9 mM, pH 4.9) for 30 min, according to the method reported by Moss^55^. The reaction was performed at 25 °C and stopped by adding 1 ml of 0.1 M sodium hydroxide. Colour development was determined at 405 nm using an Infinite F200 96-well plate reader (Tecan, Männedorf, Switzerland). Each value was the result of a series of experiments performed on 3 to 8 midguts. Statistical analyses were performed using Student’s *t*-test or ANOVA followed by Tukey’s HSD test.

### Light microscopy and transmission electron microscopy (TEM)

Midguts were dissected and fixed overnight at 4 °C in 4% glutaraldehyde in 0.1 M sodium cacodylate buffer, pH 7.2. Specimens were then postfixed in 1% osmium tetroxide for 1 h, dehydrated in an ethanol series, and embedded in an Epon/Araldite 812 mixture. Semi-thin (0.75 μm) and thin (70 nm) sections were obtained using a Leica Reichert Ultracut. Sections were stained with crystal violet and basic fuchsin and observed by using a Nikon Eclipse Ni-U microscope. Images were acquired using a Nikon DS-5M-L1 digital camera system. Thin sections were mounted on copper grids, stained using uranyl acetate and lead citrate, and observed by using a JEOL JEM-1010 electron microscope (JEOL, Tokyo, Japan). Images were acquired using a Morada digital camera (Olympus, Münster, Germany).

## Additional Information

**How to cite this article**: Romanelli, D. *et al*. Roles and regulation of autophagy and apoptosis in the remodelling of the lepidopteran midgut epithelium during metamorphosis. *Sci. Rep.* **6**, 32939; doi: 10.1038/srep32939 (2016).

## Supplementary Material

Supplementary Information
