# Supplementary material for: Roles and regulation of autophagy and apoptosis in the remodelling of the lepidopteran midgut epithelium during metamorphosis
Source: Sci Rep. 2016 Sep 9;6:32939. doi: 10.1038/srep32939 (PMC5016986; doi:10.1038/srep32939)
Supplement: Supplementary Information [file srep32939-s1.pdf]

Roles and regulation of autophagy and apoptosis in the remodelling of the lepidopteran midgut epithelium during metamorphosis

Davide Romanelli, Morena Casartelli, Silvia Cappellozza, Magda de Eguileor, and Gianluca Tettamanti

Supplementary Figure S1 - Validation of anti-Gabarap antibody.

Expression levels of BmAtg8–PE were monitored by using a commercial anti-Gabarap antibody. This antibody was selected for two reasons: i) BmAtg8 belongs to the Gabarap protein family; and ii) sequence alignment of HsGabarap (NP_009209.1) and BmAtg8 (NP_001040244.1) proteins showed a 91% identity (a). Western blot analysis demonstrated that the antibody specifically reacted with a *B. mori* Atg8 recombinant protein produced in BL21DE3/pET28+BmAtg8 bacteria and detected a 16-kDa band. By testing protein extracts obtained from silkworm midgut, the antibody recognised a 16-kDa band and a 14-kDa band that corresponded to BmAtg8 and BmAtg8–PE, respectively (b).

a

```
BmAtg8     1 MKFQYKEEHSFEKRKAEGEKIRRKYPDRVPVIVEKAPKAR  40
             |||.|||||.||||::|||||:||||||||||||||||||
HsGabarap  1 MKFVYKEEHPFEKRRSEGEKIRKKYPDRVPVIVEKAPKAR  40

BmAtg8    41 LGDLDKKKYLVPSDLTVGQFYFLIRKRIHLRPEDALFFFV  80
             :||||||||||||||||||||||||||||||.||||||||
HsGabarap 41 IGDLDKKKYLVPSDLTVGQFYFLIRKRIHLRAEDALFFFV  80

BmAtg8    81 NNVIPPTSATMGSLYQEHHDEDFFLYIAFSDENVYGN  117
             ||||||||||||.||||||:||||||||:|||:|||.
HsGabarap 81 NNVIPPTSATMGQLYQEHHEEDFFLYIAYSDESVYGL  117
```

b

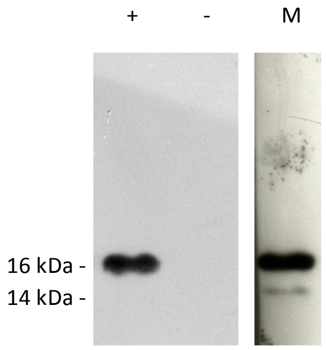

(a) Alignment of amino acid sequence of BmAtg8 and HsGabarap; (b) western blot analysis of BmAtg8 performed on BL21DE3/pET28+BmAtg8 bacteria (+), BL21DE3/pET28 bacteria (-) and silkworm midgut tissues (M), by using anti-Gabarap antibody.

Supplementary Figure S2 - RNAi-mediated inhibition of autophagy.

A) Generation of dsRNA and administration protocol

dsRNA was generated by using T7 RiboMAX™ Express RNAi System (Promega, P1700). RNA quality and integrity were assessed by electrophoresis prior to administration to the larvae.

Different conditions were assayed for dsRNA administration:

- Administration procedure (oral administration, injection into the haemocoel, injection into the haemocoel followed by electroporation)
- Stage of administration (larval stage, larva-pupa transition)
- Number of doses administered (1-6)
- Target gene (*BmATG1*, *BmATG8*)
- Amount of dsRNA (100 ng-50 μg)

Electroporation of the larvae was performed just after injecting dsRNA, by using an electric pulse generator (ECM 830, BTX, Holliston, MA, USA). The protocol was modified from Thomas[1].

B) Results

The RNAi-mediated silencing approach did not succeed in giving a strong and reproducible reduction of gene expression in larvae treated with dsRNA. A nonsignificant decrease in gene expression was observed when dsRNA was injected into the haemocoel (procedure followed or not by electroporation) (a, b). Electroporation of the larvae after dsRNA injection slightly increased the efficiency of the silencing, but this treatment led to high levels of mortality.

Unfortunately, these results are in accordance with previous and current studies on Lepidoptera, where a high variability of success in gene silencing by RNAi has been recorded, the results are conflicting, and, conversely to other insect species, a standard procedure has not been established yet[2, 3].

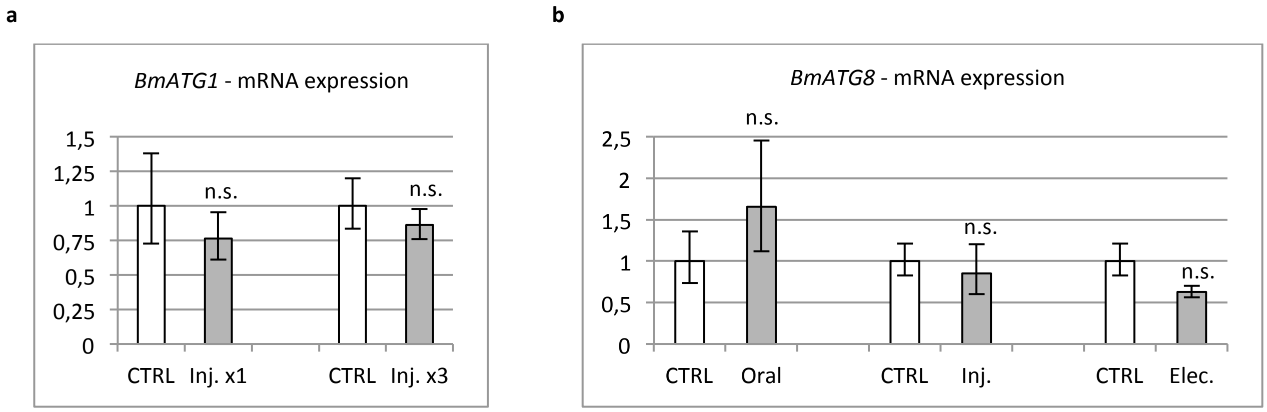

(a, b) Gene expression levels in representative RNAi experiments showing no significant difference among the treatments. (a) *BmATG1* expression 24 h following a single (Inj. ×1) or multiple injections (Inj. ×3) of *BmATG1* dsRNA during the feeding larval stage; (b) *BmATG8* expression 24 h after oral administration (Oral), injection (Inj.) or injection of dsRNA followed by electroporation (Elec.) in larvae at wandering stage.

References

1. Thomas, J.L. Electroporation, an alternative to biolistics for transfection of *Bombyx mori* embryos and larval tissues. *J. Insect Sci.* **3**, 17-28 (2003).

2. Terenius, O. *et al*. RNA interference in Lepidoptera: an overview of successful and unsuccessful studies and implications for experimental design. *J. Insect Physiol.* **57**, 231-245 (2010).

3. Kolliopoulou, A. & Swevers, L. Recent progress in RNAi research in Lepidoptera: intracellular machinery, antiviral immune response and prospects for insect pest control. *Curr. Opin. Insect Sci.* **6**, 28-34 (2014).

Supplementary Figure S3 - Evaluation of z.vad.fmk-mediated caspase inhibition.

(a) Dose-dependent inhibition of BmCaspase-1 activation obtained by treatment with z.vad.fmk at SD2 stage; (b) effect of z.vad.fmk administration at SD1 stage (25µg) on BmCaspase-1 activation.

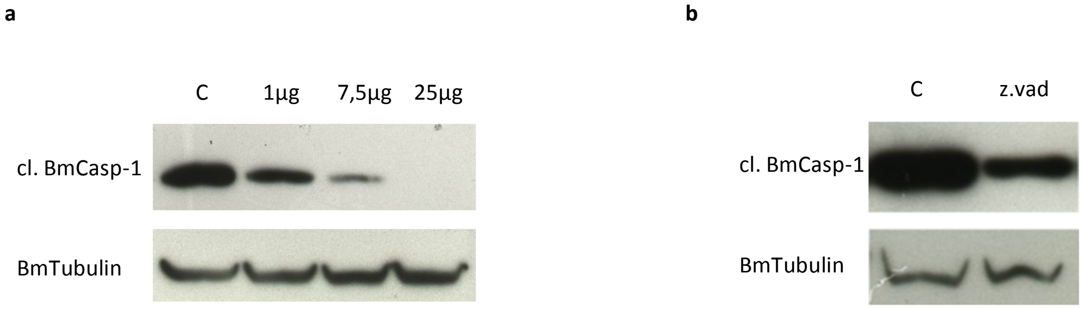

Supplementary Figure S4 - qRT-PCR analysis of *BmIAP2* and *BmIBM1* mRNA levels.

BmIAP2 (a) and *BmIBM1* (b) mRNA expression levels in the silkworm midgut during metamorphosis. Values represent mean ± s.e.m.

a

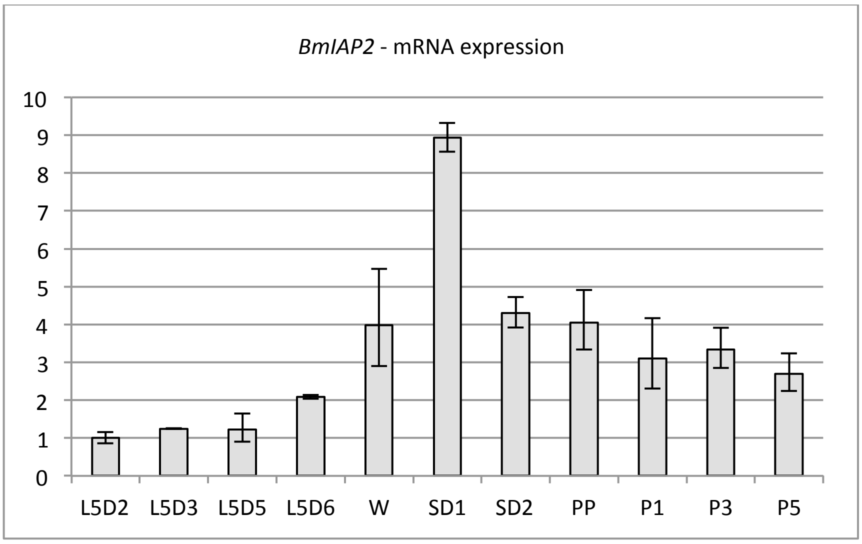

b

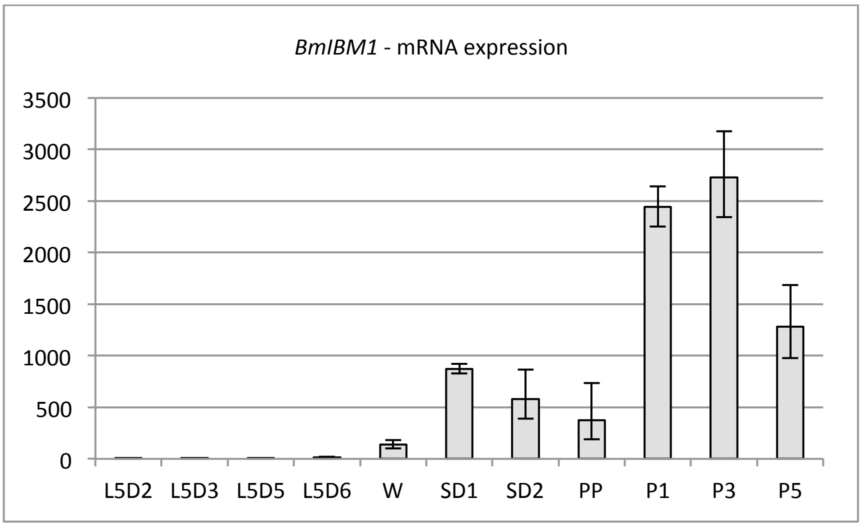

Supplementary Table S5 - Definition and description of developmental stages of the silkworm, *B. mori*, used in this study.

Stage	Definition	Larval Features	Midgut description
L5D1-L5D6	Fifth larval instar day 1–day 6	The larva actively feeds	The larval midgut epithelium is well-organized
W	Wandering stage	The larva stops feeding; spinneret pigmentation and gut purging occurs	The larval midgut epithelium is well-organized; stem cells start to proliferate
SD1	Spinning stage day 1	The larva starts spinning the cocoon	The larval midgut epithelium starts to degenerate; stem cells proliferate
SD2	Spinning stage day 2	Cocoon spinning is completed	The larval midgut epithelium detaches from the pupal epithelium; active formation of the new pupal epithelium
PP	Prepupal stage	The transition from larva to pupa occurs	The larval midgut epithelium is shed into the lumen (yellow body); the new pupal epithelium continues to grow and differentiate
P1-P9	Pupal stage day 1–day 9	Pupa	Yellow body is actively degraded; the new pupal epithelium differentiates into the adult midgut

Supplementary Table S6 - Primer sequences used in this study.

Gene Name	Accession number	Primers sequence
BmATG1	NM_001309546.1	F: CCCCGCCTATGTCTATGTTG<br>R: ATCTGATGGGTGGGAGTACG
BmATG8	NM_001046779.1	F: CCAGATCGCGTTCCTGTAAT<br>R: GAGACCCCATTGTTGCAGAT
BmCASPASE-1	NM_001043585	F: GCCTGTCGAAAGATACGCTC<br>R: CACAGCAACCAGCAGACAAT
BmCASPASE-5	NM_001195467.1	F: TCGCCATCCCGTGCTTTC<br>R: GTTGACCCCGTCCCTGTTG
BmRP49	NM_001098282.1	F: AGGCATCAATCGGATCGCTATG<br>R: TTGTGAACTAGGACCTTACGGAATC
BmIAP2	NM_001202529.1	F: CCACCAGAGACAGGCAATGA<br>R: GCCGCTGACAACACCTAAC
BmIBM1	NM_001166341.1	F: GCATAGCAGATGGAGACTGT<br>R: TTCTAACCGTGCCCTAACCA
